# A comprehensive assessment of care competence and maternal experience of first antenatal care visits in Mexico: Insights from the baseline survey of an observational cohort study

**DOI:** 10.1371/journal.pmed.1004456

**Published:** 2024-09-03

**Authors:** Svetlana V. Doubova, Claudio Quinzaños Fresnedo, Martín Paredes Cruz, Diana Perez-Moran, Ricardo Pérez-Cuevas, Verónica Meneses Gallardo, Luis Rey Garcia Cortes, Megan Carolina Cerda Mancillas, Victoria Martínez Gaytan, Miguel Angel Romero Garcia, Gilberto Espinoza Anrubio, Claudia Elsa Perez Ruiz, Carlos A. Prado-Aguilar, Augusto Sarralde Delgado, Margaret E. Kruk, Catherine Arsenault

**Affiliations:** 1 Unidad de Investigación Epidemiológica y Servicios de Salud del CMN SXXI, Instituto Mexicano del Seguro Social, Ciudad de México, México; 2 Dirección de Prestaciones Médicas, Instituto Mexicano del Seguro Social, Ciudad de México, México; 3 Division of Social Protection and Health, Interamerican Development Bank, Washington, DC, United States of America; 4 Unidad de medicina Familiar 31, Instituto Mexicano del Seguro Social, Ciudad de México, México; 5 OOAD Estado de México Oriente, Instituto Mexicano del Seguro Social, Tlalnepantla de Baz, Estado de México, México; 6 OOAD Coahuila, Instituto Mexicano del Seguro Social, Saltillo, Coahuila, México; 7 Unidad Médica de Alta Especialidad, Hospital de Gineco obstetricia N°23 Dr. Ignacio Morones Prieto, Instituto Mexicano del Seguro Social, Monterrey, Nuevo León, México; 8 Jefatura de Prestaciones Médicas, Instituto Mexicano del Seguro Social, Monterrey, Nuevo León, México; 9 OOAD DF Sur, Instituto Mexicano del Seguro Social, Ciudad de México, México; 10 OOAD Veracruz Norte, Instituto Mexicano del Seguro Social, Veracruz, México; 11 OOAD Aguascalientes, Instituto Mexicano del Seguro Social, Aguascalientes, México; 12 OOAD Jalisco, Instituto Mexicano del Seguro Social, Guadalajara, Jalisco, México; 13 Department of Global Health and Population, Harvard T.H. Chan School of Public Health, Boston, Massachusetts, United States of America; 14 Department of Global Health, Milken Institute School of Public Health, The George Washington University, Washington, DC, United States of America

## Abstract

**Background:**

Comprehensive antenatal care (ANC) must prioritize competent, evidence-based medical attention to ensure a positive experience and value for its users. Unfortunately, there is scarce evidence of implementing this holistic approach to ANC in low- and middle-income countries, leading to gaps in quality and accountability. This study assessed care competence, women’s experiences during the first ANC visit, and the factors associated with these care attributes.

**Methods and findings:**

The study analyzed cross-sectional baseline data from the maternal eCohort study conducted in Mexico from August to December 2023. The study adapted the Quality Evidence for Health System Transformation (QuEST) network questionnaires to the Mexican context and validated them through expert group and cognitive interviews with women. Pregnant women aged 18 to 49 who had their first ANC visit with a family physician were enrolled in 48 primary clinics of the Instituto Mexicano del Seguro Social across 8 states. Care competence and women’s experiences with care were the primary outcomes. The statistical analysis comprised descriptive statistics, multivariable linear and Poisson regressions.

A total of 1,390 pregnant women were included in the study. During their first ANC visit, women received only 67.7% of necessary clinical actions on average, and 52% rated their ANC experience as fair or poor. Women with previous pregnancies (adjusted regression coefficient [aCoef.] −3.55; (95% confidence intervals [95% CIs]): −4.88, −2.22, *p* < 0.001), at risk of depression (aCoef. −3.02; 95% CIs: −5.61, −0.43, *p* = 0.023), those with warning signs (aCoef. −2.84; 95% CIs: −4.65, −1.03, *p* = 0.003), common pregnancy discomforts (aCoef. −1.91; 95% CIs: −3.81, −0.02, *p* = 0.048), or those who had a visit duration of less than 20 minutes (<15 minutes: aCoef. −7.58; 95% CIs: −10.21, −4.95, *p* < 0.001 and 15 to 19 minutes: aCoef. −2.73; 95% CIs: −4.79, −0.67, *p* = 0.010) and received ANC in the West and Southeast regions (aCoef. −5.15; 95% CIs: −7.64, −2.66, *p* < 0.001 and aCoef. −5.33; 95% CIs: −7.85, −2.82, *p* < 0.001, respectively) had a higher probability of experiencing poorer care competence. Higher care competence (adjusted prevalence ratio [aPR] 1.004; 95% CIs:1.002, 1.005, *p* < 0.001) and receiving care in a small clinic (aPR 1.19; 95% CIs: 1.06, 1.34, *p* = 0.003) compared to a medium-sized clinic were associated with a better first ANC visit experience, while common pregnancy discomforts (aPR 0.94; 95% CIs: 0.89, 0.98, *p* = 0.005) and shorter visit length (aPR 0.94; 95% CIs: 0.88, 0.99, *p* = 0.039) were associated with lower women’s experience. The primary limitation of the study is that participants’ responses may be influenced by social desirability bias, leading them to provide socially acceptable responses.

**Conclusions:**

We found important gaps in adherence to ANC standards and that care competence during the first ANC visit is an important predictor of positive user experience. To inform quality improvement efforts, IMSS should institutionalize the routine monitoring of ANC competencies and ANC user experience. This will help identify poorly performing facilities and providers and address gaps in the provision of evidence-based and women-centered care.

## Abstract 2

Svetlana V. Doubova and co-workers explore antenatal care across Mexico using data collected from >1300 pregnant women who reported their individual lived experiences.

## Author summary

**Why was this study done?:**

- High-quality antenatal care (ANC) is crucial for preventing, detecting, and managing maternal and newborn complications. It involves providing comprehensive care, prioritizing evidence-based medical attention, and ensuring positive experiences for women. How healthcare providers treat women during the first ANC visit is vital in determining their satisfaction and subsequent use of health services.
- There is little evidence of the implementation of this holistic approach to ANC in Latin America and other low- and middle-income countries.

**What did the researchers do and find?:**

- The study used baseline data from the maternal eCohort in Mexico, collected through validated global QuEST network questionnaires adapted to the local context.
- The study gathered and analyzed an extensive number of user-reported experience measures and multiple health status variables to comprehensively assess women’s experiences and healthcare competency during the first ANC visit, serving as a valuable model for other Latin American and low- and middle-income countries.
- Compared to prior studies in this field, which typically gather information within a 1-year recall period, we used a 2-week data collection timeline to minimize recall bias.
- We found that higher care competence was associated with a higher likelihood of women rating their care as high quality and reporting positive experiences during the first ANC visit.
- The study’s main limitation is the potential for participants’ answers to be influenced by social desirability bias (i.e., what they think others want to hear), leading to responses they believe are socially acceptable.

**What do these findings mean?:**

- Evidence on care competence and women’s experiences should inform efforts to improve ANC quality in Mexico and Latin America.
- Care competence is an important determinant of client satisfaction.
- The gaps in care competence should be addressed to ensure positive experiences during pregnancy.

## Introduction

Women who receive continuous, supervised, quality care throughout their pregnancy, childbirth, and delivery have a 16% lower probability of neonatal death and a 24% lower probability of premature birth [1]. High-quality care improves the likelihood of preventing, detecting, and managing maternal and newborn complications in a timely manner [2]. In addition to technical care competence, women value respectful and empathetic healthcare, regardless of sociocultural and economic background [3]. How healthcare providers treat women is significant in determining their satisfaction and utilization of health services throughout the perinatal care continuum [3]. However, as many as 44% of women worldwide report experiencing negative experiences during pregnancy and childbirth care [4].

The traditional approach to obstetric care, which prioritizes the technical aspects of care and often overlooks the importance of the user–provider relationship, has been found to have adverse effects on the physical and mental health of pregnant women, leading to low levels of satisfaction, increased rates of maternal and neonatal morbidity and mortality, and a lack of trust in healthcare providers [3]. Providing evidence-based interventions and respectful antenatal and intrapartum care focusing on care quality, content of care, and women-centeredness are critical strategies to lower maternal morbidity and mortality rates [2,5].

In Mexico, gaps in clinical competence and empathetic and respectful care in the perinatal period have been reported. The 2018–2019 National Health and Nutrition Survey found that average compliance for the process of care indicators during antenatal, delivery, and postpartum care for women with obstetric risk factors was only 56.9% [6]. About 33.4% of women aged 15 to 49 experienced obstetric violence during the last childbirth [7]. There are mental health needs that should be addressed through comprehensive perinatal care. For instance, up to 20% of women develop depression during pregnancy and the postnatal period, but 75% do not receive depression care [8]. Untreated depression in pregnancy is associated with a higher probability of preterm birth, stillbirth, low birth weight, and maternal morbidity [9]. Furthermore, the high prevalence of domestic violence during pregnancy (ranging from 5.4% to 43.8% in Mexico) [10,11] puts women’s and newborns’ health at additional risk and requires appropriate care [12].

The Mexican Institute of Social Security (IMSS, Spanish acronym) is Mexico’s largest healthcare provider, with 74 million affiliated members, mainly formal labor market workers and their families [13]. Approximately 410,000 babies are born in IMSS hospitals each year. The perinatal care processes involve various healthcare providers, as stated in IMSS guidelines [14]. At primary care family medicine clinics (FMCs), family physicians and nurses are responsible for providing antenatal care (ANC), identifying obstetric risk factors, requesting laboratory and ultrasound tests, and administering vaccines and supplements. If the pregnant woman has no obstetric risks, the family doctor and nurse continue to monitor her progress. However, if there are complications or comorbidities during pregnancy, the woman must be referred to obstetrician/gynecologist specialists in second-care hospitals for further ANC. Delivery occurs in a hospital setting under the supervision of obstetrician/gynecologist specialists and neonatologists. After delivery, the woman is counter-referred to the family physician for postnatal care.

IMSS has released clinical standards for ANC. However, gaps in ANC quality remain. According to a 2014 retrospective cohort study that included 5,342 women who received ANC at IMSS, the quality of care for pregnant women was substandard [15]. Only 40.6% of women began ANC in the first trimester, and 63.5% attended 4 or more ANC visits. On average, these women received only 32.7% of the ANC recommended by IMSS clinical guidelines [15].

IMSS has recognized gaps in ANC quality and is taking steps to improve it. In March 2022, it launched the “IMSS Comprehensive Women-Centered Maternal Care Model” (AMIIMSS acronym in Spanish), a nationwide program designed to improve women’s perinatal care. AMIIMS aims to provide quality, timely, safe, and women-centered comprehensive care to women throughout their reproductive lives—from contraception to postnatal care. The program intends for healthcare to be provided in a safe and respectful environment, free of obstetric violence, and with effective communication, considering cultural differences between healthcare providers and women.

To guide the program, IMSS released technical guidelines and provided mandatory “Friendly Obstetric Care” training to hospital staff to implement the AMIIMSS model, prioritizing obstetric care improvement in hospital settings.

Although AMIIMSS’s focus is on improving obstetric care in hospitals, family physicians can voluntarily enroll in an online training program that addresses the technical aspects of ANC but does not offer person-centered care training. Additionally, there have been no change management interventions, infrastructure improvements, or process of care enhancements in FMCs as part of the AMIIMSS.

Given IMSS’s goal to improve respectful maternity care, we undertook a longitudinal eCohort study to assess women-reported experiences of care across the maternal health continuum among IMSS users. Findings from this study should provide evidence to develop actionable recommendations for decision-makers to enhance ongoing interventions aimed at delivering high-quality, comprehensive women-centered ANC.

The present study describes the adaptation and validation processes of study questionnaires in the Mexican context. Using the baseline survey, we also describe women’s characteristics, care competence, and their experience during the first ANC visit, and assess the factors associated with care competence and users’ experience.

## Methods

The study used the maternal and newborn health eCohort baseline data and questionnaires developed by the Quality Evidence for Health System Transformation (QuEST) network to longitudinally evaluate the quality of care from the women’s perspective during their pregnancy, childbirth, and postnatal period through telephone interviews [16].

### Stage 1. Adaptation and validation of the study questionnaires

The study involved adapting and validating 4 questionnaires/modules designed by the QuEST network for their use in the Mexican context. These included a baseline survey with pregnant women after their first ANC visit and follow-up surveys during pregnancy, after delivery, and postnatal periods.

The study questionnaires were translated from English to Spanish. The translation process followed the recommendations of the World Health Organization (WHO) to ensure semantic equivalence, quality, and consistency of meaning with the questionnaires’ original version. IMSS experts validated their content and conducted cognitive interviews with women during the perinatal care continuum to ensure the questionnaires were appropriate for the institutional and Mexican-culture context. The group of experts comprised 3 nurses who specialized in family medicine and maternal–child care, 2 medical doctors who were chiefs of family medicine services, 2 specialists in gynecology and obstetrics, 2 health services researchers, and a clinical psychologist, all with 10 or more years of clinical and research experience in maternal and child health. The experts reviewed and rated all questions of the 4 questionnaires based on their relevance to the study’s objectives. They also identified questions and response options difficult to understand for women and proposed clarity improvements. After 3 rounds of content validation, the experts reached a consensus on the content and wording of the questionnaire.

The content validity index (CVI) was calculated for each item, considering the expert ratings. All ratings were grouped into the following categories: relevant/very relevant, and not relevant/useful but not relevant. The CVI was calculated using the formula and methodology proposed by Lawshe and improved by Veneziano and Hooper, which quantifies the expert panel’s consensus regarding each item in the questionnaire and considers CVI equal to or greater than 0.70 as valid [17].

Two rounds of cognitive interviews were conducted with pregnant women affiliated with 2 IMSS FMCs to assess the clarity and comprehension of the study questionnaires. We applied purposive sampling to obtain a diverse schooling and pregnancy status sample and used registries of pregnant women to identify and invite women to the interviews. We completed 44 interviews, with 11 interviews per questionnaire. Of these, 12 interviews were conducted with women holding college degrees, 12 with high school graduates, 12 with secondary school graduates, and 8 with primary school education or lower. A previously elaborated guide was used to conduct the interviews.

### Stage 2. Women recruitment and baseline—First antenatal visit—Evaluation

From August 10 to December 15, 2023, pregnant women were enrolled to participate in the study.

### Selection criteria

The study included women aged 18 to 49 who attended their first ANC appointment with an IMSS family physician and signed the informed consent form. Women who planned to receive ANC and childbirth care elsewhere (for instance, private sector), those without a contact telephone number for follow-up, or those with disabilities preventing them from completing a phone survey (for instance, mental, hearing disability) were excluded from participating in the study.

### Sample size

The sample size was determined using the single population proportion formula.$n=\frac{{\left(Z\propto \right)}^{2}\;\left(p\right)\left(q\right)}{\delta^{2}}=\frac{{\left(1.96\right)}^{2}\;\left(0.68\right)\left(0.32\right)}{0.03^{2}}=\frac{\;\left(3.8416\right)\left(0.2176\right)}{0.0009}=\frac{\;\left(0.8359\right)}{0.0009}=929+40\text{\%}=929+371=1,300$. It was based on an anticipated 68% prevalence of low healthcare provider competence in the study population [15]. We also account for a 3% margin of error, 95% confidence, and a 40% potential loss during the cohort follow-up. This led to a sample of 1,300 pregnant women.

### Settings and sampling

The study included 48 FMCs across 8 Mexican states in 4 regions (North, West, Center, and Southeast). Two states were selected in each region based on their higher number of ANC visits. The states chosen were Aguascalientes and Jalisco in the West, Coahuila and Nuevo León in the North, Veracruz and Yucatán in the Southeast, and the State of Mexico and Mexico City in the Central region. Six FMCs were then selected from each state: 2 small, 2 medium, and 2 large. The size of the FMC was defined using the formula proposed by the IMSS Coordination of Information and Strategic Analysis and calculated as Total FMC affiliates / Total delegation affiliates × 100. A proportion of less than 5 was considered a small FMC, 5 to 15 a medium FMC, and more than 15 a large FMC. In each FMC, the sample of women was defined using the Lahiri method [18] based on probability proportional to the number of first-time ANC visits 4 months before the study and the goal to recruit a minimum of 163 women in each delegation to obtain 1,300 women (also see S1 and S2 Appendices files).

### Fieldwork

Women who had their first ANC appointment at study FMCs were invited by trained interviewers to participate in the study. They were informed about the study’s purpose, duration, ethical considerations (for instance, voluntary participation, potential risks and benefits, data security, and confidentiality), and contact details for the principal investigator and the IMSS Ethics Committee. All participants gave their informed consent before joining the study.

After consenting, participants could either complete a baseline questionnaire immediately or schedule a phone interview later on. Most participants (86%) preferred to conduct the baseline interviews over the phone. They often did not have time to answer the questionnaire immediately after the ANC visit but agreed to schedule a phone call for a later time. All baseline questionnaires conducted by phone were completed between the day of the first ANC visit and no more than 2 weeks afterward. The baseline interview lasted between 30 and 40 minutes. Participants received no in-kind or economic compensation for their participation in the study. For data storage and security, see S3 Appendix.

### Variables

At the baseline, information was gathered on participants’ sociodemographic and clinical characteristics, content of care, their experiences with the first ANC visit, and their ratings of the quality of healthcare they received.

The variables comprised the following:

1. The general attributes that included (i) sociodemographic characteristics such as age, level of education, occupation, and marital status; (ii) risky health behaviors such as alcohol and tobacco consumption in the last month, and (iii) Intimate partner violence measured with the violence scale and severity index validated in Mexico [19]. This scale has 19 items and evaluates psychological, physical, and sexual violence and allows calculating the severity index of intimate partner violence through the sum of the weighted scores of the 19 items, which varies from 0 to 369, where the highest score indicates greater violence.
2. Obstetric and medical history comprised the total number of pregnancies (including the current pregnancy), number of children born alive, history of abortion/miscarriage, premature birth (<37 weeks of gestation), obstetric hemorrhage, cesarean section, neonatal death; history of pregestational chronic diseases, such as previously diagnosed diabetes, prediabetes/insulin resistance, hypertension or cardiovascular disease, depression or anxiety, epilepsy, anemia, thyroid disease, respiratory diseases, gastrointestinal diseases, gynecological diseases or other chronic diseases, medication, and supplements consumption.
3. Current pregnancy and health status included weeks of gestation and trimester of pregnancy at the first antenatal visit, reasons for not seeking/receiving ANC in the first trimester, planned pregnancy, multiple pregnancies, common pregnancy discomforts (dizziness, nausea, paresthesia, or back pain); emergency warning signs: severe headache, vaginal bleeding, severe abdominal pain, difficulty breathing, fever, and others such as seizures, repeated fainting, or loss of consciousness; reduced or stopped fetal movement in the second or third trimester of pregnancy; systolic and diastolic blood pressure recorded in the woman’s health card and high blood pressure (>140/>90 mm Hg); the presence of one or more obstetric risk factors: age ≥35 years old, history of fetal death, preterm birth, pregestational diabetes, high blood pressure, current multiple pregnancies, decreased fetal movements; and self-rated health (excellent/very good, good, fair/bad). The risk of depression was evaluated with the widely used Patient Health Questionnaire (PHQ-9) validated in Mexico [20,21]. This scale has 9 questions and 4 response options ranging from 0 (none of the days) to 3 (almost every day), with an overall total between 0 and 27. The severity of the symptoms is organized into 6 risk categories of depression: 0 to 4 (minimal), 5 to 9 (mild), 10 to 14 (moderate), 15 to 19 (moderate to severe), and 20–27 (severe) [22]. For this study and considering the low prevalence of cases with a risk of depression ≥10, the last 3 categories were grouped into a single one of moderate to severe risk.
4. Content of care during the first ANC visit comprised waiting time and visit duration and the following clinical actions during the visit, including history taking: assessing the date of the last menstruation, taking history about previous pregnancies, (including asking about miscarriages, stillbirths, premature births, previous cesarean sections, or neonatal deaths), assessing the presence of warning signs during the current pregnancy; history of pregestational chronic diseases and their treatments, including mental health disorders. Examinations, laboratory and imaging studies, and preventive care included measurements of blood pressure, weight, height, and fetal heartbeat (for women with ≥12 weeks of gestation); referral to a general urine test, full blood count testing for anemia screening, rapid test for HIV, glucose measurement for diabetes detection, syphilis testing; ultrasound scan before 24 weeks; referral for tetanus vaccination in unvaccinated women or those with more than 10 years since the last immunization. Provision of information about the number of babies expected, the delivery date, the delivery plan, the possibility, and reasons for needing a cesarean section. Counseling during the first ANC visit about nutrition, physical exercise during pregnancy, situations that cause stress, quitting risky health behaviors such as smoking and/or drinking alcohol in women with these habits, and information on where to seek help in case of intimate partner violence in women who suffered violence, and counseling about common pregnancy discomforts and signs of emergency. Referral to a consultation or follow-up with a specialist in obstetrics and gynecology and scheduling the next antenatal appointment.
5. Women’s experiences during the first ANC visit were measured by asking women to rate 8 components of their care on a scale from 1 to 5 (poor, fair, good, very good, and excellent): (i) knowledge and skills of their healthcare provider; (ii) the level of respect showed by the provider; (iii) availability of medical equipment or access to lab tests; (iv) clarity of the provider’s explanations; (v) degree to which the provider involved women in decisions about their care; (vi) courtesy and helpfulness of the healthcare facility staff, other than healthcare provider; (vii) the amount of time the provider spent with the woman and (viii) the waiting time.
6. We also created 2 summary variables. **(6.1) A summative measure of care competence** was defined as the percentage of clinical actions performed by the healthcare provider during the first ANC visit out of the total number of actions the woman required based on her medical and obstetric history. To construct this variable, we considered all previously mentioned clinical actions, including taking an obstetric and medical history, physical examinations, laboratory tests and imaging, preventive care, and counseling. (6.2) **A user experience score** was defined as the sum of the response scores of the 8 user experience indicators described in the fifth subsection.

### Statistical analysis (also see S4 Appendix)

We first conducted descriptive analyses of the study population, the content of care, and women’s experiences during the first antenatal visit. The categorical variables were presented as percentages, while numerical variables with a normal distribution were presented as mean and standard deviation; numerical variables without normal distribution were presented as median with minimum and maximum.

Second, we investigated the factors associated with care competence using a multivariable linear regression model. Our modeling strategy was based on VanderWeele and Shpitser criterion for confounder selection [23,24]. These authors recommend including all conceptually and clinically relevant covariates to ensure the final model adjusts for even slight confounding (and is not subject to potential *p*-value hacking). Through a literature review, we identified relevant sociodemographic obstetric and medical history variables that previous studies linked to user experiences, satisfaction with healthcare, perception of the quality of care, and providers’ competence. Finally, we analyzed factors associated with better user experience during the first antenatal visit. For this purpose, we considered the distribution of the user experience score and conducted multivariable Poisson regression analyses, including all conceptually or clinically relevant covariates in the model and considering the health facilities as a cluster variable. In addition, 19% of women had missing data for the user experience score or other variables.

To avoid bias related to the missing data in the participants’ responses, we corrected this by fitting the final multivariable Poisson regression model using stabilized inverse probability weights (IPWs) [25]. The denominator of stabilized IPWs was the probability of “having missing data” given the available covariates without missing data. These covariates were the participants’ age, education, presence of risky health behaviors, chronic disease, and number of pregnancies. The numerator was the probability of “having missing data” regardless of the covariates. The IPW approach tends to be simpler than multiple imputation (MI) because, unlike MI, which requires the creation and analysis of multiple imputed datasets, IPW involves only a single weighted dataset. IPW can provide a straightforward way to obtain unbiased estimates if the model for the missing data mechanism is correctly specified [26]. Results from the regression analysis without IPWs are provided in S5 Appendix.

Before performing the multivariable regression analyses, we estimated crude coefficients through bivariate linear regression and crude prevalence ratios (PRs) through bivariate Poisson regression (S6 and S7 Appendices). We also confirmed the absence of multicollinearity and interactions among the study covariates. In addition, the standard errors of both regression models were adjusted for the clustered sampling approach. We used the unique ID of the FMCs where women received ANC to adjust the standard errors. A *p*-value of ≤0.05 was considered statistically significant.

We analyzed data using the statistical software Stata 14 (Stata Corp LP; College Station, Texas). The study is reported as per the Strengthening the Reporting of Observational Studies in Epidemiology (STROBE) guideline (S8 Appendix).

### Ethics approval

The study was approved by the IMSS National Research and Ethics Committees (R-2022-785-064). Before participating in the study, all women signed the informed consent form.

## Results

### Stage 1

As part of the adaptation process, all 4 study questionnaires underwent changes to enhance the questions’ clarity, sequence, and relevance. In each questionnaire, irrelevant questions were removed, and unclear questions were improved to ensure better understanding. For example, 7 questions were eliminated in the first questionnaire as they did not apply to the health providers activities during the first ANC visit in IMSS FMCs. Additionally, 16 questions and 8 response options were modified to improve clarity. Moreover, questions related to partner violence were replaced with questions from a validated scale in Mexico.

### Stage 2

A total of 1,555 pregnant women were invited to participate in a study after their first ANC visit. Out of those, 1,390 women (89.4%) agreed to participate. The 2 main reasons for not participating were lack of time (66.1%) and lack of interest because they would not receive any in-kind or economic incentive (33.9%). When comparing the characteristics of those who agreed and those who did not, we found that the women who declined to participate had higher education levels than those who agreed (64.2% of acceptors had high school or higher education, while 76.2% of non-acceptors had high school or higher education).

### Participants’ characteristics

A total of 1,390 women participated in the study (Table 1). Most were between 18 and 34 years old (86.4%), with high school or higher (64.2%), and had a paid job (61.0%). Only 3.8% reported consuming alcoholic beverages and 1.7% smoking. The majority lived in a common law union (43.7%) or were married (38.4%); 9.6% reported suffering from intimate partner violence, with psychological violence being the most frequent (8.3%) and the severity of violence being mild, with a median of 6 and a range between 4 and 127 points.

**Table 1:** Demographic characteristics and obstetric and medical history of the IMSS maternal eCohort participants.

Variables	Total *n* = 1,390
**I. General characteristics**	**n (%)**
**Age groups**	
18–34 years	1,201 (86.4)
≥35 years	189 (13.6)
**Education**	
Primary school degree or lower	66 (4.8)
Complete secondary school	429 (30.9)
High school with or without university degree	893 (64.2)
Did not answer	2 (0.1)
**Occupation**	
Remunerated job	848 (61.0)
Housewife/unemployed/student	542 (39.0)
**Risky health behaviors**	
Alcohol consumption	52 (3.8)
Tobacco consumption	24 (1.7)
Alcohol and/or tobacco consumption	72 (5.2)
**Marital status**	
Married or partnered	1,140 (82.1)
Single/divorced/separated/widow	223 (16.0)
Did not answer	27 (1.9)
**Suffering some type of intimate partner violence**	133 (9.6)
Psychological violence	115 (8.3)
Physical violence	32 (2.3)
Sexual violence	25 (1.8)
**II. Obstetric history**	
Primigravida	509 (36.6)
**Among women with a previous pregnancy:**	*n* = 881
Miscarriage/stillborn baby	73 (8.3)
Premature birth (<37 weeks of gestation)	67 (7.6)
Obstetric hemorrhage	33 (3.7)
Cesarean section	347 (39.4)
Neonatal death	21 (2.4)
**III. Medical history**	*n* = 1,390
History of any of the below pregestational chronic disease(s)	264 (19.0)
Depression/anxiety	78 (5.6)
Hypertension or cardiovascular disease	37 (2.7)
Diabetes/prediabetes/insulin resistance	37 (2.7)
Thyroid disease	36 (2.6)
Gynecological disease	30 (2.2)
Respiratory diseases	18 (1.3)
Gastrointestinal diseases	12 (0.9)
Anemia	11 (0.8)
Epilepsy	9 (0.7)
Other pregestational chronic disease(s)	44 (3.2)
Consumption of medicines and supplements	472 (34.0)

Of all the women surveyed, 36.6% had their first pregnancy, while the remaining 63.4% had already experienced pregnancy. Among those who had previous pregnancies, 8.3% had reported having a miscarriage, 7.6% had experienced premature birth, 3.7% had obstetric hemorrhage, 39.4% had undergone cesarean section, and 2.4% had to face neonatal death. As for medical history, 19% reported having a chronic illness before pregnancy. The top 5 medical diagnoses were depression or anxiety (5.6%), followed by high blood pressure and diabetes/prediabetes (2.7% both), thyroid disease (2.6%), and gynecological diseases (2.2%). Additionally, 34% mentioned taking medication.

Regarding the current pregnancy, 45.8% began ANC during the first trimester, 42.5% during the second trimester, and 11.7% during the third trimester. The main reasons for not initiating ANC during the first trimester were not knowing they were pregnant (61.2%), not having social security at the beginning of pregnancy (28.2%), or not finding available appointments (12.5%). Only 48.8% reported planning their pregnancy, and multiple pregnancies were reported by 1.2%; 73.2% experienced common pregnancy discomforts, such as dizziness, nausea, or back pain, while 35.8% reported experiencing some emergency warning signs. The most frequent emergency symptoms were headache (22.5%), vaginal bleeding (11.0%), and severe abdominal pain (9.3%). Of the women with blood pressure recorded (*n* = 800), 6.9% had elevated blood pressure. According to the institutional guideline, 26.9% presented one or more obstetric risk factors. Most women rated their health as good or excellent, while only 16.3% rated it as fair or poor. The evaluation of the depression risk with the PHQ9 scale revealed that 15.7% had a mild risk of depression, while 3.4% had a moderate to severe risk of depression (Table 2).

**Table 2:** Characteristics of the current pregnancy and health status among IMSS maternal eCohort participants.

Variable	Total *n* = 1,390
**I. Current pregnancy**	**n (%)**
Initiation of ANC	
First trimester	637 (45.8)
Second trimester	591 (42.5)
Third trimester	162 (11.7)
Reasons for not seeking/receiving ANC in the first trimester among those who sought care in the second or third trimester (*n* = 753):	
Did not know she was pregnant	461 (61.2)
Did not have social security	212 (28.2)
Tried to come earlier, but no appointments were available, or another problem for getting the appointment came up	94 (12.5)
Long waiting time	44 (5.8)
Other	80 (10.6)
Planned pregnancy	678 (48.8)
Multiple pregnancy	16 (1.2)
Common pregnancy discomforts (dizziness, nausea, etc.)	1,017 (73.2)
Number of emergency warning signs	
0	892 (64.2)
1	316 (22.7)
≥2	182 (13.1)
Type of warning sign	
Severe headache	313 (22.5)
Vaginal bleeding	153 (11.0)
Severe abdominal pain	129 (9.3)
Difficulty breathing	92 (6.6)
Fever	51 (3.7)
Others (seizures, repeated fainting, or loss of consciousness)	14 (1.0)
Stopped fetal movement (among women in the second/third trimester of pregnancy *n* = 753)	7 (0.9)
High blood pressure (>140/>90 mm Hg) (among women with register of the blood pressure in their health card *n* = 800)	55 (6.9)
Presence of one or more obstetric risk factors^‡^	374 (26.9)
**II. Current health status**	
Self-rated health	
Fair/Poor	226 (16.3)
Risk of depression	
Minimal risk 0–4	1,125 (80.9)
Mild risk 5–9	218 (15.7)
Moderate to severe ≥10	47 (3.4)

^‡^Presence of one or more obstetric risk factors according to the institutional guideline: age ≥35 years old, history of fetal death, preterm birth, pregestational diabetes, high blood pressure, current multiple pregnancy, decreased/stopped fetal movements.

### Care during the first ANC visit

The median waiting time for the first ANC visit was 30 minutes (range 0 to 540) and the median duration of the visit was 20 minutes. Only 20.9% waited less than 15 minutes. Only half reported that their first ANC visit lasted 20 minutes or longer, while 16.7% had a visit lasting less than 15 minutes (Table 3).

**Table 3:** Competent care: First ANC visit duration and content of care.

Variables	Total *n* = 1,390	Variables	Total *n* = 1,390
**I. Waiting time and duration**	**n (%)**	**III. Examinations and referrals**	**n (%)**
Waiting time, minutes		Blood pressure recorded in a personal health card	800 (57.6)
Median (minimum-maximum)	30 (0–540)		
Waiting time		Weight measurement	1,352 (97.3)
<15 minutes	291 (20.9)	Height measurement	1,135 (81.7)
15–29 minutes	283 (20.4)	Fetal heartbeat measurement^‡^	*n* = 841 447 (53.2)
30–60 minutes	478 (34.4)		
>60 minutes	338 (24.3)		
Visit duration, minutes		Referral to a general urine test	*n* = 1,390 1,302 (93.7)
Median (minimum-maximum)	20 (3–120)		
Visit duration		Referral to a full blood count testing for anemia screening	1,293 (93.0)
<15 minutes	232 (16.7)		
15–19 minutes	380 (27.3)		
20–29 minutes	480 (34.6)	Referral to a glucose measurement for DM screening	1,088 (78.3)
≥30 minutes	298 (21.4)	Referral to a rapid HIV screening	1,210 (87.1)
**II. First antenatal visit content** **IIa. Obstetric history taking regarding:**		Referral to a syphilis testing	1,023 (73.6)
The first day of the last menstrual period	1,361 (97.9)	Ultrasound scan before 24 weeks	*n* = 1,141 623 (54.6)
Previous pregnancies	*n* = 881 70 (7.9)	Referral for tetanus vaccination^‡‡^	*n* = 1,012 598 (59.1)
Miscarriage or stillborn baby	*n* = 73 70 (95.9)	**IV. Information on**	*n* = 1,172
Preterm birth	*n* = 67 9 (13.4)	Number of babies expected, (in women after 8 weeks of gestation)	506 (43.2)
Previous cesarean sections	*n* = 347 23 (6.6)	Delivery date	*n* = 1,390 1,102 (79.3)
Neonatal death	*n* = 21 3 (14.3)	Delivery plan	403 (29.0)
Warning symptoms of the current pregnancy	*n* = 1,390 668 (48.1)	Possibility of having a cesarean section	178 (12.8)
**IIb. Taking medical history**		**V. Counseling**	
Diabetes	*n* = 29 22 (75.9)	Nutrition	805 (57.9)
Hypertension and/or cardiovascular diseases	*n* = 37 25 (69.4)	Physical exercise during pregnancy	612 (44.0)
Mental health disorders in women with a previous diagnosis of depression	*n* = 78 15 (19.2)	Warning signs	1,004 (72.2)
Mood in women who reported feeling depressed (PHQ ≥6 points)	*n* = 173 36 (20.8)	Quitting risky health behaviors (smoking and/or drinking alcohol	*n* = 72 52 (72.2)
Medications	*n* = 472 361 (76.5)	Common pregnancy discomforts	*n* = 1,017 436 (42.9)
**III. Examinations and referrals**	***n* = 1,390**	Where to seek help in case of intimate partner violence	*n* = 133 7 (5.3)
Blood pressure measurement	1,295 (93.2)	Things that cause stress	233 (16.8)
**VI. Referrals and follow-up**	*n* = 1,390	**VI. Referrals and follow-up**	*n* = 1,390
Referral to a consultation or follow-up with a specialist in obstetrics and gynecology at the second care level	430 (30.9)	Scheduling the next antenatal appointment	1,334 (96.0)
**Healthcare competence**			
**Mean (SD)**			67.7 (13.4)
Median (minimum-maximum)			68.4 (12–100)
**Poor healthcare competence** (<60% clinical care activities performed from those required), %			369 (26.6)

^‡^Fetal heartbeat measurement (in women with ≥12 weeks of gestation).

^‡‡^Referral for tetanus vaccination in unvaccinated women or those with more than 10 years after the last vaccination.

Regarding the clinical actions during the first ANC visit, in most cases (97.9%), the physicians asked women about the date of their last menstruation. However, only 7.9% of women were asked about their previous pregnancy. Among these women, 95.9% were asked if they had experienced miscarriages and stillborn babies. Only 13.4% were asked about their history of preterm births. Only 6.6% of women with a previous cesarean section and only 14.3% of women with a previous neonatal death were asked about this history. Out of the total number of women, only 48.1% were asked about warning symptoms during their current pregnancy.

A relatively high percentage of women with diabetes (76%) and hypertension (69.4%) were asked about their medical history by their physicians, as opposed to only 19.2% of women with previously diagnosed depression. Furthermore, only 20.8% of women at risk of depression were asked about their mood, and 76.5% were asked about their medication consumption.

Out of the women surveyed, 93.2% reported having their blood pressure measured, but only 57.6% had it recorded on their personal health cards; 97.3% reported that their weight was measured, while 81.7% reported that their height was measured. Half of the women (53.2%) with ≥12 weeks of gestation reported that their physician measured the fetal heartbeat. Additionally, 93.7% were referred to a general urine test, 93% for a blood test, 87.1% for the rapid HIV test, 78.3% for rapid glucose measurement to screen for diabetes, and 73.6% for the Venereal Diseases Research Laboratory measurement for syphilis detection. Only 54.6% of the women were referred to ultrasound scan before 24 weeks, and 59.1% of women not vaccinated against tetanus or those with more than 10 years after the last vaccination were referred to tetanus vaccination. Less than half (43.2%) of women who were 8 or more weeks pregnant were informed by their physicians about the number of babies they were carrying, while 79.3% received information about the probable delivery date. Only 29.0% were informed about their birth plans, and just 12.8% were told about the possibility of having a cesarean section. The main reasons for the cesarean section were a previous one (53.9%) or the mother’s health issues (25.3%).

Regarding health-related counseling, 57.9% of women were counseled about nutrition, 44% about physical activity, and 16.8% about stress management. Also, 72.2% of women with a history of smoking or alcohol use were advised to quit these habits. Only 5.3% of women who reported experiencing intimate partner violence received information about where to seek help. Also, 72.2% of women were counseled about warning signs of emergency, and 42.9% of women received recommendations and treatment to address common discomforts during pregnancy. Four hundred and thirty women (30.9%) were referred to a gynecologist for consultation or follow-up, and 96% had the next antenatal visit scheduled.

On average, only 67.7% of the necessary clinical actions were performed based on women’s medical and obstetric history. Overall, 26.6% of women received poor care competence, with less than 60% of activities performed.

The 3 main reasons for receiving ANC at the IMSS FMC were healthcare covered by social security and being affiliated with a specific FMC (57.5%), proximity to home (19.8%), or need for maternity leave (6.3%), while only 4% said it was due to the good skills of IMSS physicians.

**Fig 1:**
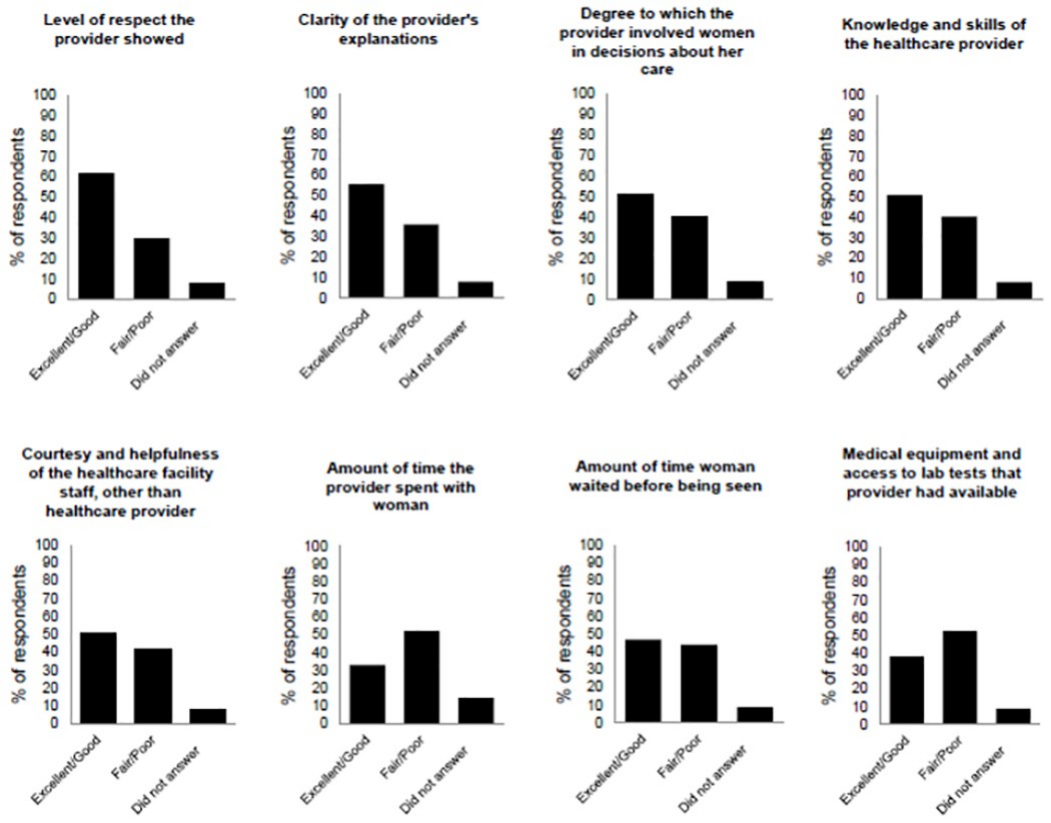
Experiences of women during the first antenatal visit.

When specifically asked about women’s perceptions of different aspects of the first ANC visit, the results showed that 30% of women rated the level of respect they received from their physician as regular or poor, and 36.1% rated the clarity of the explanations as regular or poor. Additionally, about 40% rated the degree to which the physician involved them in decision-making about their care and the physician’s knowledge and skills in caring for pregnant women as fair or poor. Furthermore, 41.8% rated the courtesy and friendliness of the healthcare facility staff as fair or poor, while 52.1% rated the time the provider spent with them as fair or poor. Finally, 52.7% rated the availability of equipment and lab tests as fair or poor (Fig 1).

Overall user experience during the first ANC visit, defined as the sum of the 8 previously reported indicators, ranging from a score of 8 (poor experiences) to 40 points (excellent experience), revealed that, on average, women scored only 23 points, indicating general regular experience.

Table 4 presents the factors associated with care competence score during the first ANC visit. Women’s characteristics, such as multiple pregnancies, risk of depression, warning signs, common pregnancy discomforts, and initiating ANC in the first or second trimester, were associated with poorer care competence during the first ANC visit. Additionally, receiving ANC in the West and Southeast regions or having an ANC consultation length under 20 minutes were associated with lower competence scores.

**Table 4:** Results of a multivariable linear regression for the factors associated with the care competence score during the first ANC visit (*n* = 1,363).

	Adjusted Coefficient	Clustered Robust Std. Err	95% CI	*p*
**General characteristics and medical history**				
Age ≥35 years	−0.53	1.38	−3.32, 2.25	0.702
Single/Divorced/Separated/Widow	1.12	0.93	−0.74, 2.99	0.233
Remunerated job	−0.04	0.59	−1.22, 1.14	0.944
Education				
Primary school degree or lower	−1.94	1.55	−4.80, 0.93	0.181
Complete secondary school	0.07	0.87	−1.67, 1.81	0.936
Risky health behaviors	−1.68	1.18	−4.07, 0.70	0.162
Fair or poor self-rated health	0.27	0.99	−1.72, 2.26	0.784
Pregestational chronic diseases	0.19	0.96	−1.75, 2.13	0.843
**Current pregnancy**				
Multigravida	−**3.55**	**0.66**	−**4.88,** −**2.22**	**<0.001**
Risk of depression	−**3.02**	**1.29**	−**5.61,** −**0.43**	**0.023**
Common pregnancy discomforts	−**1.91**	**0.94**	−**3.81,** −**0.02**	**0.048**
Warning signs	−**2.84**	**0.90**	−**4.65,** −**1.03**	**0.003**
One or more obstetric risk factors	1.99	1.03	−0.07, 4.06	0.059
Initiation of ANC				
First trimester	−**4.24**	**1.33**	−**6.92,** −**1.56**	**0.003**
Second trimester	−**2.59**	**1.12**	−**4.85,** −**0.33**	**0.025**
**Health facility location, size, and duration of the first antenatal visit**				
Region				
Central	−2.55	1.43	−5.42, 0.33	0.081
West	−**5.15**	**1.24**	−**7.64,** −**2.66**	**<0.001**
Southeast	−**5.33**	**1.25**	−**7.85,** −**2.82**	**<0.001**
Size of the clinic where women received their first ANC visit				
Small	-2.22	1.97	-6.18, 1.73	0.264
Large	-0.54	0.98	-2.51, 1.42	0.582
Duration of first antenatal visit				
<15 minutes	−**7.58**	**1.31**	−**10.21,** −**4.95**	**<0.001**
15–19 minutes	−**2.73**	**1.02**	−**4.79,** −**0.67**	**0.010**
20–29 minutes	1.83	1.04	−3.93, 0.27	0.086

R-squared = 0.1392. Std. Err, standard error. 95% CI, confidence interval. Reference values: 18–34 years old; married/common union; housewife/student/unemployed; high school with or without university degree; without risky health behaviors; perception of health as good, very good, or excellent; without chronic disease; primigravida; without risk of depression; no common pregnancy discomforts; no warning signs; no obstetric risk factors; beginning of ANC in the third trimester; North region; medium clinic size; duration of the first antenatal consultation ≥30 minutes.

Table 5 shows that higher care competence and the size of the clinic (small) were variables associated with better user experience, while common pregnancy discomforts and visit length of less than 15 minutes were associated with lower ***women’s*** overall experience score during the first ANC visit.

**Table 5:** Results of an IP-weighted multivariable Poisson regression analysis for the factors associated with women’s experiences during the first ANC visit (*n* = 1,123).

	Adjusted Prevalence Ratio	Clustered Robust Std. Err	95% CI	*P*
**Care competence**	**1.004**	**<0.001**	**1.002, 1.005**	**<0.001**
**Other covariates** **General characteristics and medical history**				
Age ≥35 years	1.04	0.04	0.97, 1.11	0.227
Single/Divorced/Separated/Widow	0.99	0.03	0.93, 1.05	0.741
Remunerated job	0.98	0.02	0.95, 1.02	0.394
Education				
Primary school degree or lower	0.93	0.05	0.85, 1.03	0.183
Complete secondary school with or without high school	0.99	0.02	0.95, 1.02	0.531
Risky health behaviors	1.04	0.04	0.96, 1.13	0.329
Fair or poor self-rated health	1.00	0.02	0.96, 1.04	0.960
Pregestational chronic diseases	1.00	0.02	0.96, 1.04	0.991
**Current pregnancy**				
Multigravida	1.01	0.02	0.97, 1.04	0.629
Risk of depression	1.00	0.02	0.95, 1.04	0.889
Common pregnancy discomforts	**0.94**	**0.02**	**0.89, 0.98**	**0.005**
Warning signs	1.01	0.02	0.97, 1.05	0.615
One or more obstetric risk factors	0.97	0.02	0.93, 1.01	0.181
Initiation of ANC				
First trimester	1.03	0.03	0.98, 1.09	0.221
Second trimester	0.97	0.02	0.93, 1.02	0.311
**Health facility location, size, and duration of the first antenatal visit**				
Region				
Central	1.03	0.07	0.90, 1.17	0.694
West	1.04	0.06	0.92, 1.17	0.494
Southeast	0.93	0.05	0.83, 1.05	0.259
Size of the clinic where women received their first ANC visit				
Small	**1.19**	**0.07**	**1.06, 1.34**	**0.003**
Large	1.09	0.05	0.99, 1.20	0.055
Duration of first antenatal visit				
<15 minutes	**0.94**	**0.03**	**0.88, 0.99**	**0.039**
15–19 minutes	0.97	0.03	0.92, 1.03	0.348
20–29 minutes	0.99	0.02	0.94, 1.04	0.614

Std. Err, standard error. 95% CI, confidence interval. Care competence measured as a percentage of activities performed by the healthcare providers during the first antenatal visit regarding the required activities based on women’s medical and obstetric history. Reference values: 18–34 years old; married/common union; housewife/student/unemployed; high school with or without university degree; without risky health behaviors; perception of health as good, very good, or excellent; without chronic disease; primigravida; without risk of depression; no common pregnancy discomforts; no warning signs; no obstetric risk factors; beginning of ANC in the third trimester; North region; medium clinic size; duration of the first antenatal consultation ≥30 minutes.

## Discussion

The study comprehensively assessed provider competence and women’s experiences during the first ANC visit across 48 IMSS primary care clinics in 8 states of Mexico. The key findings show that, on average, women received less than 70% of the necessary clinical actions, indicating suboptimal health providers’ proficiency. Several characteristics were associated with poorer care competence, including women’s health status and obstetric history, shorter visit length, and being in the West or South East regions of Mexico. The findings also show that almost half of the pregnant women rated their experience during the first ANC visit as fair or poor. Higher care competence and receiving care in a small clinic as compared to a medium size clinic were factors associated with a better first ANC visit experience, while common pregnancy discomforts and shorter visit length were associated with lower women’s experience.

The first ANC visit is important for identifying obstetric risks and building trusting relationships between women and healthcare providers to ensure positive pregnancy outcomes. Comprehensive ANC is essential to achieve these objectives and ensure pregnant women’s and their babies’ well-being [2]. This type of care should prioritize competent, evidence-based medical attention and produce positive experiences for women. Therefore, assessing the quality of ANC should include measuring the experiences and opinions of women who receive it. Unfortunately, this holistic approach to ANC and its evaluation is not widely implemented, leading to significant information gaps and missed opportunities for improvement.

Provider competency in ANC refers to the knowledge and implementation of institutional evidence-based practice guidelines and WHO recommendations on ANC tailored to women’s needs [2]. A competent provider should be able to conduct thorough maternal and fetal assessment and counseling. IMSS has evidence-based practice guidelines, but health providers do not use them regularly, and there are no clear metrics to ascertain their use. Our results indicate deficient obstetric and medical history taking, physical examinations, and laboratory and ultrasound screenings. For instance, less than 1 in 10 women who had been pregnant before were asked about their previous pregnancies, and less than half were screened for warning symptoms during the current pregnancy. Additionally, only half of the women received a referral for an ultrasound scan before 24 weeks of gestation. These clinical activities are essential to identify and manage high-risk pregnancies and to prevent complications during childbirth. For instance, previous studies have indicated that women who have had 2 or more miscarriages face twice the risk of experiencing very preterm delivery, placenta previa, preterm premature rupture of membranes, and low birth weight [27]. Moreover, women with a history of fetal death are at high risk for subsequent pregnancy loss with less than 1 in 4 pregnancies resulting in infant survival [28]. At the same time, routine second-trimester ultrasound before 24 weeks can enhance the detection of major fetal abnormalities and increase the number of women opting for termination of pregnancy due to this reason [29].

Counseling about obstetric danger signs would help identify and treat severe pregnancy complications in a timely manner [2], yet in our study, only 7 in 10 women received counseling for danger signs. However, it was documented that in developing countries like Mexico, pregnant women have low to medium awareness of obstetric danger signs [30]. This lack of awareness can lead to delays in seeking urgent healthcare [31]. Providing counseling on warning signs during ANC visits can increase the likelihood of recognizing pregnancy danger signs by 8 times [32]. Therefore, healthcare providers should prioritize counseling on obstetric warning signs.

It is also important to counsel pregnant women about unhealthy habits and provide nutrition and physical activity advice to ensure healthy pregnancy outcomes. A meta-analysis of 34 studies on the effect of nutrition counseling targeting maternal diet and supplement intakes during pregnancy revealed significant improvements in health outcomes. The interventions led to a 30% reduction in the risk of anemia in late pregnancy, a 105 g increase in birth weight, and a 19% decrease in the risk of preterm delivery [33]. Furthermore, dietary and physical activity interventions and a combination of both were also associated with a 26% reduction in the risk of preeclampsia and a 9% reduction in cesarean section [34,35]. However, in the present study, only 4 out of 10 women received physical activity counseling, and 6 out of 10 received nutritional counseling, revealing the need to reinforce these activities.

Existing studies on ANC provider competency, including counseling, are primarily from low-income countries in Africa and Asia [33–35]. In Latin America, research on healthcare provider competency in ANC is scarce. Studies mainly focus on the adequacy of care in terms of the timing of ANC initiation, the number of attended appointments, and antenatal procedures, reporting that the percentage of women who received adequate ANC ranges from 21% to 71% in different settings [36–40].

In our study, we found that better care competence was strongly correlated with better women’s experiences. This suggests that pregnant women are able to recognize good quality care. Gathering women’s feedback about their initial ANC visit can offer significant insights and valuable information. Therefore, a comprehensive assessment of ANC should include feedback from women about their first ANC visit, including the provider’s level of knowledge and respect, the clarity of the information given, the involvement of the woman in decision-making, the courtesy of the staff, the duration of the visit, and the availability of necessary equipment. These characteristics of care are essential to ensure women-centered care and to encourage women to continue using ANC [41]. However, most research in Latin America and other low- and middle-income countries focuses on women’s satisfaction [42–46]. It lacks detailed information on specific experiences that could help identify gaps in care and design improvement strategies. In our study, lower-rated experiences included limited access to lab tests and medical equipment, long wait times before being seen, and short provider–patient interactions. Additionally, women reported perceived gaps in the knowledge and skills of healthcare providers and a lack of involvement in decisions about their care. Importantly, higher healthcare providers’ competence was associated with better women’s overall opinions about their experiences during the first antenatal visit. However, we identified that care competence varied across states, with the Southeast (Veracruz and Yucatán) and the West (Aguascalientes and Jalisco) regions showing lower provider competency than the Northern region (Coahuila and Nuevo León). Variations in the quality of maternal care at IMSS across states were previously reported, revealing low quality in both poor and wealthy states [47]. Additionally, women’s experiences varied based on the size of FMCs, with better experiences in small FMCs compared to medium-sized ones. Similar findings were observed in the United Kingdom [48,49], where small clinics provided more personalized care. This variation in care emphasizes the need for standardizing IMSS care across the country to ensure consistent quality of care across facilities and states.

The association between poor ANC competence and women’s overall opinions of their experiences during the first ANC visit underscores the need to address systemic barriers contributing to this issue. Institutional, structural, and provider-related barriers must be identified and addressed to implement evidence-based practices in primary care.

From an institutional perspective, common barriers include shortages of healthcare providers, heavy workloads, and time constraints [50]. Several studies at IMSS have reported these barriers, emphasizing the need for strengthening human resources [51,52]. For example, family physicians at IMSS work 6-hour shifts with up to 24 patients, allowing an average of 15 minutes per patient consultation. However, our study suggests that to better meet women’s specific needs, the first ANC visit should be at least 20 to 30 minutes long. This duration ensures sufficient time for necessary assessments and counseling, thereby creating positive experiences for women.

Another barrier is the lack of providers’ competency and monitoring of user experiences. IMSS should institutionalize regular monitoring and feedback of ANC providers’ competency and women’s experiences at FMCs. Monitoring these indicators could serve as benchmarks for performance against best practices and offer succinct feedback to health providers. This could help identify and address gaps between evidence-based practices and the care women receive. Incorporating pregnant women’s feedback about the ANC they receive, tailoring ANC to their needs, and ensuring their preferences and values are considered could be pivotal in developing a women-centered model of ANC. Additionally, the information gathered from monitoring can be integral in devising simulation-based training and e-learning platforms customized to address the identified educational needs of health providers delivering ANC.

Regarding healthcare providers, common barriers include inadequate training, and insufficient knowledge and skills related to evidence-based practices, poor communication skills, and unclear delineation of roles and responsibilities. Implementing comprehensive training strategies for primary care teams—including physicians, nurses, social workers, and nutritionists—is crucial. These strategies should focus on enhancing providers’ technical, communication, and user-centered skills, as suggested by the present study findings and existing literature on this topic [50]. These strategies should prioritize the provision of appropriate assessment and counseling to pregnant women, especially those who have had previous pregnancies, who are experiencing warning signs, and who are at risk of depression or suffering from domestic violence. These recommendations align with the WHO and IMSS ANC guidelines [2,14]. The training should focus on underperformed activities in the Southeast and West regions where lower care competence and women’s experiences were reported. This is especially important as a recent study revealed that the user’s overall perception of public healthcare providers’ low quality is associated with a higher likelihood of private sector use [53]; therefore, if women do not receive competent person-centered ANC at IMSS, they will choose to use private sector care, which could lead to their catastrophic health expenditures.

Additionally, all members of the primary care teams should educate women on danger signs, diet, and physical health during pregnancy, childbirth, and the postnatal period to raise women’s awareness of the importance of early identification and treatment of danger signs, as well as healthy behaviors for the health of the mother and her baby.

The present study has several strengths and limitations. One of its strengths is the comprehensive evaluation of the first ANC visit, including both patient-reported experience and healthcare competency measures. Our study also benefited from adapting and validating the questionnaires designed by international experts from the QuEST network for global use. The translated version of this questionnaire was content validated by experts from IMSS, and patient input was taken into account during the questionnaire codevelopment through cognitive interviews. Another strength is the large sample of women from 8 Mexican states and a 2-week timeline for data collection to avoid recall bias, as previous studies collected information over a year or more after the healthcare visit took place.

Nevertheless, our study has limitations. First, the content of care was self-reported by participants who may not be able to accurately report what was done during the visit. More educated or multipara women may report specific ANC clinical items better. Some measures (for instance, blood pressure) were extracted from maternal health cards, but these cards were often incomplete. There is also a possibility that participants’ ratings of their experiences can be prone to social desirability bias when participants provide responses that they think are socially acceptable; however, the physical distance between the interviewer and respondent, along with the respondent’s inability to see nonverbal signs of the interviewer, can lead to more sincere responses. The cross-sectional analysis of baseline information does not allow causal inference. There was also a high prevalence of missing data for the user experience score, which we addressed using stabilized IPWs. Moreover, the present paper only addresses the first ANC visit. Additional clinical care items may be performed in follow-up visits. Finally, the study only focused on pregnant women who received ANC at IMSS and did not include other public healthcare providers in Mexico. This limits the generalizability of the findings. However, it is worth noting that IMSS is the largest health institution in Mexico, providing healthcare to over 57% of the national population.

In conclusion, care competence during the first ANC visit is an important predictor of positive user experience. Improvements in care competence and respectful and patient-centered care are needed for pregnant women at IMSS. Our study provides evidence on the attributes of ANC that need to be improved, calling for action from stakeholders, including policymakers, healthcare providers, and researchers.

## Supporting information

S1 Appendix Protocol sections on the sampling method and data collection procedures. (DOCX)

S2 Appendix Flowchart of the sampling process. (DOCX)

S3 Appendix Data storage and security. (DOCX)

S4 Appendix Predefined analysis plan. (DOCX)

S5 Appendix Regression analysis without IPWs. (DOCX)

S6 Appendix Results of a bivariate linear regression for the factors associated with the care competence score during the first ANC visit. (DOCX)

S7 Appendix Results of an IP-weighted bivariate Poisson regression analysis for the factors associated with women’s experiences during the first ANC visit. (DOCX)

S8 Appendix STROBE checklist. (DOC)

## Abbreviations

95% CI: 95% confidence interval
aCoef.: adjusted regression coefficient
AMIIMSS: IMSS Comprehensive Women-Centered Maternal Care Model
ANC: antenatal care
aPR: adjusted prevalence ratio
CVI: content validity index
FMC: family medicine clinic
IMSS: Mexican Institute of Social Security
IPW: inverse probability weight
MI: multiple imputation
PR: prevalence ratio
QuEST: Quality Evidence for Health System Transformation
WHO: World Health Organization
