# Supplementary material for: A comprehensive assessment of care competence and maternal experience of first antenatal care visits in Mexico: Insights from the baseline survey of an observational cohort study
Source: PLoS Med. 2024 Sep 3;21(9):e1004456. doi: 10.1371/journal.pmed.1004456 (PMC11371229; doi:10.1371/journal.pmed.1004456)
Supplement: S1 Appendix — (DOCX) [file pmed.1004456.s001.docx]

**S1. Appendix.** **Protocol sections on sampling method and data collection procedures**

**Sampling method**

We conducted convenience non-probability sampling. First, from 4 regions of the country (North, West, Center, and Southeast), we selected 2 states per region that had the family medicine clinics (FMCs) with the higher number of antenatal care (ANC) visits. The states chosen were Aguascalientes and Jalisco in the West, Coahuila and Nuevo León in the North, Veracruz and Yucatán in the Southeast, and the State of Mexico and Mexico City in the Central region. Second, in each state, we selected six FMCs: two small, two medium, and two large. The size of the FMC was defined using the formula proposed by the IMSS Coordination of Information and Strategic Analysis and calculated as Total FMC affiliates/ Total delegation affiliates x 100. A proportion of less than five was considered a small FMC, 5 to 15 a medium FMC, and more than 15 a large FMC. When selecting the FMCs, we also considered that three of six FMCs (one small, one medium, and one large) in each state have the same referral hospital with high adherence to "IMSS Comprehensive Women-Centered Maternal Care Model" and another three FMCs have the referral hospital with low adherence to "IMSS Comprehensive Women-Centered Maternal Care Model", as defined by the program authorities. Third, in each FMC, the sample of women was defined using the Lahiri method (Maskurul, et al., 2015) based on probability proportional to the number of first ANC visits four months before the study and the goal to recruit a minimum of 163 women in each delegation to obtain 1,300 women (also see S2 Appendix with a flowchart of the sampling process). In each FMC, half of the sample will be recruited during the morning shift and the other half during the afternoon shift. If the sample size is odd, an additional woman will be recruited in the morning shift. To achieve the required sample size at each clinic, all consecutive women who meet the inclusion criteria will be invited to participate in the study.

**Data collection procedures**

The study will collect information using four electronic questionnaires/modules designed by the QuEST network and adopted by the researchers for use in the Mexican context, and located on a secure password-protected website developed specifically for the study. These include a baseline survey of pregnant women after their first ANC visit and follow-up surveys during pregnancy, after delivery, and during the postnatal period. These questionnaires will be administered by trained interviewers.

The interviewers will invite women attending their first ANC appointment at study FMCs to participate in the study. The interviewers will inform women about the study's purpose, duration, ethical considerations (e.g., voluntary participation, potential risks and benefits, data security, and confidentiality), and contact details of the principal investigator and the IMSS Ethics Committee.

Those women who agree to participate and sign the informed consent form will be invited to either complete a baseline questionnaire immediately or schedule a phone interview within the following two-week timeframe. The baseline questionnaire will collect information on participants' sociodemographic and clinical characteristics, content of care, experiences with the first antenatal care visit, and ratings of the quality of healthcare they received.

After entering the study and answering the baseline questionnaire, women will be contacted monthly to answer monthly follow-up phone surveys during the prenatal period. These interviews will collect data on health status, health services utilization patterns, content, experiences, quality of care, and women's satisfaction with prenatal care.

After giving birth, women will be contacted again to answer the questionnaire about the quality of delivery or cesarean section care received, experience with respectful care, health status, including mother and newborn complications. This survey will be administered once between 2 and 4 weeks after delivery.

Finally, women will be invited to answer a follow-up telephone survey in the postnatal period. This survey will assess the woman's postpartum health, the health of the newborn, care-seeking patterns (for the mother and newborn), postnatal care visits, experience, and satisfaction with postnatal care. It will be administered once between 6 and 8 weeks after delivery.

.
