# Supplementary material for: A comprehensive assessment of care competence and maternal experience of first antenatal care visits in Mexico: Insights from the baseline survey of an observational cohort study
Source: PLoS Med. 2024 Sep 3;21(9):e1004456. doi: 10.1371/journal.pmed.1004456 (PMC11371229; doi:10.1371/journal.pmed.1004456)
Supplement: S2 Appendix — (DOCX) [file pmed.1004456.s002.docx]

**Regions**

**Regions**

Sampling

**West**

(7 states)

2022 mean number of ANC visits

96,644

**North**

(11 states)

2022 mean number of ANC visits

93,896

**Southeast**

(8 states)

2022 mean number of ANC visits

43,188

**Center**

(6 states)

2022 mean number of ANC visits

80,596

**Aguascalientes**

2022

number of ANC visits

49,086

**Jalisco**

2022

number of ANC visits

246,070

**Coahuila**

2022

number of ANC visits

122,050

**Nuevo León**

2022

number of ANC visits

181,612

**Veracruz**

2022

number of ANC visits

63,680

**Mexico City**

2022

number of ANC visits

105,865

**Yucatán**

2022

number of ANC visits

55,208

**Lihiri**

**Method**

**State of Mexico**

2022 number of ANC visits 180,557

[Escriba una cita del documento o el resumen de un punto interesante. Puede situar el cuadro de texto en cualquier lugar del documento. Use la ficha Herramientas de dibujo para cambiar el formato del cuadro de texto de la cita.]

2

Number of patients per clinic based on probability proportional to the number of first-time ANC visits four months before the study in each clinic.

Two small FMCs

Two medium FMCs

Two large FMCs
