# Supplementary material for: A comprehensive assessment of care competence and maternal experience of first antenatal care visits in Mexico: Insights from the baseline survey of an observational cohort study
Source: PLoS Med. 2024 Sep 3;21(9):e1004456. doi: 10.1371/journal.pmed.1004456 (PMC11371229; doi:10.1371/journal.pmed.1004456)
Supplement: S3 Appendix — (DOCX) [file pmed.1004456.s003.docx]

**S3. Appendix. Data storage and security**

The information was gathered using electronic questionnaires on a website (https://amiimss.com/). A hosting service from a licensed company was hired to ensure data privacy and protection. The company has a privacy policy that complies with the Federal Law on the Protection of Personal Data. The hosting service provided SSL (Secure Sockets Layer) encryption to verify the website's identity through an encrypted https:// address with a secure padlock in the browser. Additionally, CAPTCHA images (Completely Automated Public Turing test to tell Computers and Humans Apart) were used to ensure the privacy and security of the information. The collected information was stored weekly in the principal investigator's office computer and was password protected.
