# Supplementary material for: A comprehensive assessment of care competence and maternal experience of first antenatal care visits in Mexico: Insights from the baseline survey of an observational cohort study
Source: PLoS Med. 2024 Sep 3;21(9):e1004456. doi: 10.1371/journal.pmed.1004456 (PMC11371229; doi:10.1371/journal.pmed.1004456)
Supplement: S4 Appendix — (DOCX) [file pmed.1004456.s004.docx]

### **S4. Appendix. Predefined analysis plan.**

**Evaluation of baseline data form the maternal eCohort in Mexico on care competence and women's experience during the first antenatal care visit.**

**Statistical analysis plan**

First, we will conduct descriptive analyses of the study population comprising (i) the sociodemographic variables, including age, level of education, occupation, and marital status; (ii) the risky health behaviors included alcohol and tobacco consumption in the last month; (iii) intimate partner violence; (iv) obstetric and medical history; (v) characteristics of current pregnancy and women health status; (vi) content of care, and women’s experiences during the first antenatal visit. The categorical variables will be presented as percentages. After checking for normality of numerical variables using Shapiro–Wilk test and histogram, the numerical variables with a normal distribution will be presented as mean and standard deviation; numerical variables without normal distribution will be presented as median, minimum and maximum and interquartile ranges. We will also check variables with missing data and calculate the percentage of missing values for each variable.

Second, we will investigate the factors associated with care competence (dependent variable). The analysis will include univariable and multivariable regression models that will be chosen considering the distribution of the care competence score. Our modeling strategy of the multivariable regression model will be based on VanderWeele and Shpitser (2011) criterion for confounder selection [1,2]. These authors recommended including all conceptually and clinically relevant covariates to ensure the final model adjusts for even slight confounding (and is not subject to potential p-value hacking). Therefore, we will include in the multivariable regression model all relevant sociodemographic, obstetric and medical history variables that previous studies linked to user experiences, satisfaction with healthcare, perception of the quality of care, and providers' competence and which we identified through the literature review. In addition, the standard errors of the regression model will be adjusted for family medicine clinic (FMC) clusters using the unique ID of each FMC where women were cared for.

Third, we will analyze factors associated with user experience during the first antenatal visit (dependent variable). The analysis will include univariable and multivariable regression models that will be chosen considering the distribution of the user experience score. We will adhere to the same modeling strategy and adjust the standard errors of the regression model for FMC clusters using the unique ID of each FMC.

In addition, if there is the missing data more than 10% in any of the study variables that we include in the multivariable regressions, to avoid bias related to the missing data in the participants' responses, we will correct this by fitting the final multivariable Poisson regression model using stabilized inverse probability weights (IP-weights) [3]. The denominator of stabilized IP weights will be the probability of “having missing data” given the available covariates without missing data. The numerator will be the probability of “having missing data” regardless of the covariates.

Before performing the multiple regression analyses, we will confirm the absence of multicollinearity and interactions among the study covariates. A p-value of ≤0.05 will be considered statistically significant. We will analyze data using the statistical software Stata 14 (Stata Corp LP; College Station, TX).

**References**

1. VanderWeele TJ. Principles of confounder selection. Eur J Epidemiol. 2019;34(3):211–219.
2. VanderWeele TJ, Shpitser I. A new criterion for confounder selection. Biometrics. 2011;67(4):1406–1413.
3. Hernan MA, Robins JM. Causal Inference: What If. Boca Raton: Chapman & Hall/CRC; 2020.
