# Supplementary material for: A comprehensive assessment of care competence and maternal experience of first antenatal care visits in Mexico: Insights from the baseline survey of an observational cohort study
Source: PLoS Med. 2024 Sep 3;21(9):e1004456. doi: 10.1371/journal.pmed.1004456 (PMC11371229; doi:10.1371/journal.pmed.1004456)
Supplement: S5 Appendix — (DOCX) [file pmed.1004456.s005.docx]

**S5 Appendix.**

**Results of the multivariable Poisson regression analysis for the** **factors associated with** **women’s experiences during the first ANC visit without using IPWs (n=1123)**

|  | **Adjusted RP** | **Robust**  **Std. Err** | **95% CI** | **P** |
| --- | --- | --- | --- | --- |
| **Care competence** | **1.004** | **0.001** | **1.002-1.005** | **<0.001** |
| **Other covariates**  **General characteristics and medical history** |  |  |  |  |
| Age ≥ 35 years | 1.04 | 0.04 | 0.98, 1.11 | 0.220 |
| Single/Divorced /Separated/Widow | 0.99 | 0.03 | 0.93, 1.05 | 0.758 |
| Remunerated job | 0.98 | 0.02 | 0.95, 1.02 | 0.391 |
| Education  Primary school degree or lower  Complete secondary school | 0.93  0.99 | 0.05  0.02 | 0.85, 1.03  0.96, 1.02 | 0.185  0.534 |
| Risky health behaviors | 1.04 | 0.04 | 0.96, 1.13 | 0.324 |
| Fair or poor self-rated health | 1.00 | 0.02 | 0.96-1.04 | 0.990 |
| Pre-gestational chronic diseases | 1.00 | 0.02 | 0.96, 1.04 | 0.998 |
| **Current pregnancy** |  |  |  |  |
| Multigravida | 1.01 | 0.02 | 0.97, 1.04 | 0.632 |
| Risk of depression | 1.00 | 0.02 | 0.95, 1.04 | 0.897 |
| Common pregnancy discomforts | **0.94** | **0.02** | **0.89, 0.98** | **0.005** |
| Warming signs | 1.01 | 0.02 | 0.97, 1.05 | 0.628 |
| One or more obstetric risk factors | 0.97 | 0.02 | 0.93, 1.01 | 0.176 |
| Initiation of antenatal care  First trimester  Second trimester | 1.03  0.97 | 0.03  0.02 | 0.97, 1.09  0.93, 1.02 | 0.220  0.313 |
| **Health facility location, size and duration of the first prenatal visit** |  |  |  |  |
| Region  Central  West  Southeast | 1.03  1.04  0.93 | 0.07  0.06  0.06 | 0.90, 1.17  0.92, 1.17  0.83, 1.05 | 0.697  0.498  0.254 |
| Size of the clinic where women received their first prenatal care visit  small  large | **1.19**  1.09 | **0.07**  0.05 | **1.06, 1.34**  0.99-1.20 | **0.003**  0.054 |
| Duration of first prenatal visit  <15 minutes  15 - 19 minutes  20 - 29 minutes | **0.94**  0.97  0.99 | **0.03**  0.03  0.02 | **0.88, 0.99**  0.92, 1.03  0.94, 1.04 | **0.038**  0.359  0.627 |

Care competence measured as a percentage of activities performed by the healthcare providers during the first prenatal visit regarding the required activities based on women's medical and obstetric history. Reference values: 18-34 years old; married/common union; housewife/student/ unemployed; high school with or without university degree; without risky health behaviors; perception of health as good, very good, or excellent; without chronic disease; primigravida; without risk of depression; no common pregnancy discomforts; no warning signs; no obstetric risk factors; beginning of prenatal care in the third trimester; North region; medium clinic size; duration of the first prenatal consultation ≥30 minutes.
