# Supplementary material for: A comprehensive assessment of care competence and maternal experience of first antenatal care visits in Mexico: Insights from the baseline survey of an observational cohort study
Source: PLoS Med. 2024 Sep 3;21(9):e1004456. doi: 10.1371/journal.pmed.1004456 (PMC11371229; doi:10.1371/journal.pmed.1004456)
Supplement: S6 Appendix — (DOCX) [file pmed.1004456.s006.docx]

**S6 Appendix. Results of a bivariate linear regression for the factors associated with the care competence score during the first ANC visit**

|  | **Crude**  **Coefficient** | **Clustered Robust**  **Std. Err** | **95%CI** | **p** |
| --- | --- | --- | --- | --- |
| **General characteristics and medical history** |  |  |  |  |
| Age ≥ 35 years | -0.34 | 1.21 | -2.77, 2.09 | 0.779 |
| Single/Divorced /Separated/Widow | 1.64 | 0.89 | -0.16, 3.44 | 0.073 |
| Remunerated job | 0.13 | 0.74 | -1.36, 1.61 | 0.865 |
| Education  Primary school degree or lower  Complete secondary school | -2.60  -1.16 | 1.82  1.03 | -6.26, 1.07  -3.24, 0.91 | 0.160  0.265 |
| Risky health behaviors | -2.99 | 1.58 | -6.16, 0.19 | 0.065 |
| Fair or poor self-rated health as | **-2.65** | **1.09** | **-4.85, -0.45** | **0.019** |
| Pre-gestational chronic diseases | -1.53 | 1.16 | -3.88, 0.81 | 0.194 |
| **Current pregnancy** |  |  |  |  |
| Multigravida | **-4.21** | **0.73** | **-5.69, -2.73** | **<0.001** |
| Risk of depression | **-5.50** | **1.28** | **-8.09, -2.91** | **<0.001** |
| Common pregnancy discomforts | **-3.65** | **1.05** | **-5.76, -1.54** | **0.001** |
| Warning signs | **-4.64** | **1.04** | **-6.73, -2.55** | **<0.001** |
| One or more obstetric risk factors | 0.32 | 0.84 | -1.36, 2.00 | 0.702 |
| Initiation of antenatal care  First trimester  Second trimester | **-5.28**  **-2.63** | **1.46**  **1.23** | **-8.21, -2.35**  **-5.11, -0.14** | **0.001**  **0.039** |
| **Health facility location, size and duration of the first antenatal visit** |  |  |  |  |
| Region  Central  West  Southeast | -1.91  **-5.96**  **-4.08** | 1.77  **1.38**  **1.27** | -5.47, 1.65  **-8.73, -3.19**  **-6.63, -1.53** | 0.285  **<0.001**  **0.002** |
| Size of the clinic where women received their first antenatal care visit  small  large | -2.39  -0.51 | 2.30  1.52 | -7.01, 2.23  -3.57, 2.54 | 0.303  0.736 |
| Duration of first antenatal visit  <15 minutes  15 - 19 minutes  20 - 29 minutes | **-7.34**  **-2.37**  -1.07 | **1.21**  **1.12**  1.01 | **-9.78, -4.89**  **-4.63, -0.11**  -3.10, 0.96 | **<0.001**  **0.040**  0.293 |

Std. Err: standard error. 95%CI: Confidence interval. Reference values: 18-34 years old; married/common union; housewife/student/ unemployed; high school with or without university degree; without risky health behaviors; perception of health as good, very good, or excellent; without chronic disease; primigravida; without risk of depression; no common pregnancy discomforts; no warning signs; no obstetric risk factors; beginning of antenatal care in the third trimester; North region; medium clinic size; duration of the first antenatal consultation ≥30 minutes.
