# Supplementary material for: A comprehensive assessment of care competence and maternal experience of first antenatal care visits in Mexico: Insights from the baseline survey of an observational cohort study
Source: PLoS Med. 2024 Sep 3;21(9):e1004456. doi: 10.1371/journal.pmed.1004456 (PMC11371229; doi:10.1371/journal.pmed.1004456)
Supplement: S7 Appendix — (DOCX) [file pmed.1004456.s007.docx]

**S7 Appendix. Results of an IP-weighted bivariate Poisson regression analysis for the** **factors associated with** **women’s experiences during the first ANC visit (n=1123)**

|  | **Crude**  **Prevalence Ratio** | **Clustered Robust**  **Std. Err** | **95% CI** | **P** |
| --- | --- | --- | --- | --- |
| **Care competence** | **1.004** | **0.001** | **1.002, 1.005** | **<0.001** |
| **Other covariates**  **General characteristics and medical history** |  |  |  |  |
| Age ≥ 35 years | 1.01 | 0.03 | 0.96, 1.07 | 0.714 |
| Single/Divorced /Separated/Widow | 1.00 | 0.03 | 0.94, 1.06 | 0.958 |
| Remunerated job | 0.99 | 0.02 | 0.96, 1.03 | 0.698 |
| Education  Primary school degree or lower  Complete secondary school | 0.94  0.99 | 0.05  0.02 | 0.85, 1.03  0.96, 1.02 | 0.198  0.492 |
| Risky health behaviors | 1.04 | 0.05 | 0.96, 1.14 | 0.327 |
| Fair or poor self-rated health | 0.98 | 0.02 | 0.94, 1.02 | 0.356 |
| Pre-gestational chronic diseases | 1.00 | 0.02 | 0.96, 1.04 | 0.963 |
| **Current pregnancy** |  |  |  |  |
| Multigravida | 0.98 | 0.01 | 0.96, 1.01 | 0.273 |
| Risk of depression | 0.99 | 0.03 | 0.93, 1.04 | 0.612 |
| Common pregnancy discomforts | **0.92** | **0.02** | **0.88, 0.97** | **0.001** |
| Warning signs | 0.99 | 0.02 | 0.95, 1.03 | 0.632 |
| One or more obstetric risk factors | 0.98 | 0.02 | 0.94, 1.02 | 0.366 |
| Initiation of antenatal care  First trimester  Second trimester | 1.01  0.96 | 0.03  0.03 | 0.95, 1.07  0.91, 1.01 | 0.816  0.136 |
| **Health facility location, size and duration of the first antenatal visit** |  |  |  |  |
| Region  Central  West  Southeast | 1.02  1.01  0.92 | 0.07  0.07  0.06 | 0.90, 1.16  0.88, 1.16  0.82, 1.04 | 0.711  0.842  0.185 |
| Size of the clinic where women received their first antenatal care visit  small  large | **1.20**  1.09 | **0.07**  0.05 | **1.08, 1.35**  0.99, 1.19 | **0.001**  0.063 |
| Duration of first antenatal visit  <15 minutes  15 - 19 minutes  20 - 29 minutes | **0.93**  0.97  0.98 | **0.03**  0.03  0.03 | **0.87, 0.99**  0.92, 1.02  0.93, 1.03 | **0.021**  0.243  0.501 |

Std. Err: standard error. 95%CI: Confidence interval. Care competence measured as a percentage of activities performed by the healthcare providers during the first antenatal visit regarding the required activities based on women's medical and obstetric history. Reference values: 18-34 years old; married/common union; housewife/student/ unemployed; high school with or without university degree; without risky health behaviors; perception of health as good, very good, or excellent; without chronic disease; primigravida; without risk of depression; no common pregnancy discomforts; no warning signs; no obstetric risk factors; beginning of antenatal care in the third trimester; North region; medium clinic size; duration of the first antenatal consultation ≥30 minutes.
