# Supplementary material for: A comprehensive assessment of care competence and maternal experience of first antenatal care visits in Mexico: Insights from the baseline survey of an observational cohort study
Source: PLoS Med. 2024 Sep 3;21(9):e1004456. doi: 10.1371/journal.pmed.1004456 (PMC11371229; doi:10.1371/journal.pmed.1004456)
Supplement: S8 Appendix — (DOC) [file pmed.1004456.s008.doc]

STROBE Statement—checklist of items that should be included in reports of observational studies

|  | | Item No | | Recommendation | | | Item location in the manuscript | |
| --- | --- | --- | --- | --- | --- | --- | --- | --- |
| **Title and abstract** | | 1 | | (*a*) Indicate the study’s design with a commonly used term in the title or the abstract | | | First paragraph of the “Methods and findings” section of the Abstract. | |
|  |  |  |  | (*b*) Provide in the abstract an informative and balanced summary of what was done and what was found | | | First paragraph of the “Methods and findings” section of the Abstract. | |
| Introduction | | | | | | |  |  |
| Background/rationale | | 2 | | Explain the scientific background and rationale for the investigation being reported | | | The first eight paragraphs of the “Introduction” section. | |
| Objectives | | 3 | | State specific objectives, including any prespecified hypotheses | | | Last paragraph of the “Introduction” section. | |
| Methods | | | | | | |  |  |
| Study design | | 4 | | Present key elements of study design early in the paper | | | First paragraph of the “Methods” section. | |
| Setting | | 5 | | Describe the setting, locations, and relevant dates, including periods of recruitment, exposure, follow-up, and data collection | | | First sentence of the description of stage 2 and first paragraph of “Setting and sampling” sub-section of the “Methods” section. | |
| Participants | | 6 | | *Cross-sectional study*—Give the eligibility criteria, and the sources and methods of selection of participants | | | Complete information (one paragraph) of the “Selection criteria” subsection of the description of stage 2 of the “Methods” section. | |
| Variables | | 7 | | Clearly define all outcomes, exposures, predictors, potential confounders, and effect modifiers. Give diagnostic criteria, if applicable | | | Complete information (7 paragraphs) of the “Variables” subsection of the description of stage 2 of the “Methods” section. | |
| Data sources/ measurement | | 8* | | For each variable of interest, give sources of data and details of methods of assessment (measurement). Describe comparability of assessment methods if there is more than one group | | | Information of the “Variables” subsection of the description of stage 2 of the Methods section and S2 Appendix. | |
| Bias | | 9 | | Describe any efforts to address potential sources of bias | | | Third paragraph of the “Statistical analysis” subsection | |
| Study size | | 10 | | Explain how the study size was arrived at | | | The paragraph of the sample size subsection of the “Methods” section. | |
| Quantitative variables | | 11 | | Explain how quantitative variables were handled in the analyses. If applicable, describe which groupings were chosen and why | | | The first four paragraphs of the “Statistical analysis” subsection | |
| Statistical methods | | 12 | | (*a*) Describe all statistical methods, including those used to control for confounding | | | The first four paragraphs of the “Statistical analysis” subsection | |
|  | |  | | (*b*) Describe any methods used to examine subgroups and interactions | | | The fourth paragraph of the “Statistical analysis” subsection | |
|  | |  | | (*c*) Explain how missing data were addressed | | | The third paragraph of the “Statistical analysis” subsection | |
|  | |  | | (*d*) *Cross-sectional study*—If applicable, describe analytical methods taking account of sampling strategy | | | The fourth paragraph of the “Statistical analysis” subsection | |
|  | |  | | (*e*) Describe any sensitivity analyses | | | Last sentence of the third paragraph of the “Statistical analysis” subsection | |
| Results | |  | |  | | |  |  |
| Participants | | 13* | | (a) Report numbers of individuals at each stage of study—eg numbers potentially eligible, examined for eligibility, confirmed eligible, included in the study, completing follow-up, and analysed | | | The second paragraph of the “Results” section | |
|  | |  | | (b) Give reasons for non-participation at each stage | | | The second paragraph of the “Results” section | |
|  | |  | | (c) Consider use of a flow diagram | | | N/A |  |
| Descriptive data | | 14* | | (a) Give characteristics of study participants (eg demographic, clinical, social) and information on exposures and potential confounders | | | The third, fourth, and fifth paragraphs of the “Results” section and Tables 1 and 2 | |
|  | |  | | (b) Indicate number of participants with missing data for each variable of interest | | | Tables 1 to 3 and Figure 1 | |
| Outcome data | | 15* | | *Cross-sectional study—*Report numbers of outcome events or summary measures | | | Paragraphs 6 to 12of the “Results” section and Table 3 | |
| Main results | | 16 | | (*a*) Give unadjusted estimates and, if applicable, confounder-adjusted estimates and their precision (eg, 95% confidence interval). Make clear which confounders were adjusted for and why they were included | | | Tables 4 and 5 | |
|  | |  | | (*b*) Report category boundaries when continuous variables were categorized | | | N/A |  |
|  | |  | | (*c*) If relevant, consider translating estimates of relative risk into absolute risk for a meaningful time period | | | Not relevant |  |
|  | |  | |  | | |  |  |
| Other analyses | 17 | | | | Report other analyses done—eg analyses of subgroups and interactions, and sensitivity analyses | N/A | | |
| Discussion | | | | | |  | | |
| Key results | 18 | | Summarise key results with reference to study objectives | | | The first paragraph of the “Discussion” section | | |
| Limitations | 19 | | Discuss limitations of the study, taking into account sources of potential bias or imprecision. Discuss both direction and magnitude of any potential bias | | | The penultimate paragraph of the “Discussion” section | | |
| Interpretation | 20 | | Give a cautious overall interpretation of results considering objectives, limitations, multiplicity of analyses, results from similar studies, and other relevant evidence | | | Paragraphs 3 to 7 of the “Discussion” section | | |
| Generalisability | 21 | | Discuss the generalisability (external validity) of the study results | | | The last two sentences of the “Discussion” section, before the conclusion | | |
| Other information | | | | | |  | | |
| Funding | 22 | | Give the source of funding and the role of the funders for the present study and, if applicable, for the original study on which the present article is based | | | The source of funding is provided at the end of the manuscript. | | |
